# The Entrepreneur’s Multiple Identities Dynamic Interaction and Strategic Entrepreneurial Behavior: A Case Study Based on Grounded Theory

**DOI:** 10.3390/bs13020167

**Published:** 2023-02-14

**Authors:** Qing Yin, Gang Liu

**Affiliations:** School of Business, East China University of Science and Technology, Shanghai 200237, China

**Keywords:** strategic entrepreneurship, entrepreneur, identity, well-being, family firm

## Abstract

Family firms face many uncertainties in a dynamic entrepreneurial context. Previous studies have shown strategic entrepreneurship can help firms effectively cope with uncertainties. There are few studies on how family-firm entrepreneurs make strategic decisions and engage in strategic entrepreneurial behavior. This may prevent managers lacking the necessary action guidelines from effectively implementing entrepreneurial behavior. we aim to explore the micro-behavioral mechanisms of strategic entrepreneurship in family firms. A longitudinal single case study based on grounded theory was conducted to explore these issues. Results show that (i) the entrepreneur’s identity will constantly evolve to adapt to the entrepreneurial context during the life cycle of the corporation; (ii) entrepreneurs influence strategic entrepreneurial activities through the dynamic evolution and interaction of their identities; (iii) different entrepreneurial emotional states affect the strategic entrepreneurial behavior mechanisms. As a result, dynamic interactions between entrepreneurial identities have a significant impact on emotional states such as entrepreneurial well-being, which may significantly affect the implementation pattern of strategic entrepreneurial behaviors and the sustainable development of the firm. This paper provides a novel theoretical perspective on the path and behavioral choices of strategic entrepreneurship in firms, and also provides action guidelines and theoretical references for family business managers to implement strategic entrepreneurial behavior.

## 1. Introduction

Family firms are characterized by family members owning the majority of the enterprise, some family members being involved or indirectly involved in the daily management of the enterprise, and the family having the important decision-making powers of the enterprise [1]. The managers of these companies have more freedom to act unilaterally and idiosyncratically, so the behavior of such a company is clearly influenced by the characteristics of the family managers and owners [2]. After more than 40 years of reform and opening-up, China’s marketing progress has been accelerating, and in this process, family enterprises, as important participants and builders of the market economy, have acquired unprecedented development. The technology-based family firm is a typical representative of a family firm, which means that the family members master the core technologies of product production and can use these technologies to produce products with excellent quality and broad market appeal, which are difficult for competitors to imitate or surpass in a short time [3]. The “Industry 4.0” and “Made in China 2025” strategies put forward intelligent manufacturing as an important direction to promote the transformation and upgrading of manufacturing industries. Based on the conceptual framework of “Industry 4.0” intelligent manufacturing system, intelligent design, intelligent processing, intelligent control, and other capabilities directly promote the intelligent upgrading and rapid development of technology-based family firms. According to statistics, by 2021, China’s high-tech enterprises had reached 330,000, and the proportion of private high-tech enterprises had reached 83%. Family firms account for more than 80% of private high-tech enterprises. Today, technology-based family firms are mainly distributed in the fields of new energy, new materials, AI, medical devices, electronic communications, and so on. Technology-based family firms have become an important force to promote scientific and technological innovation, increase employment and improve people’s livelihoods.

Internationalization, industry 4.0, technological iteration, digital change, inequality, regional wars, and anti-globalization competition have together defined the environmental context in which we live today. In particular, on 11 March 2020, the World Health Organization declared the highly transmissible coronavirus disease COVID-19 a pandemic, signaling its global spread. The public health tactic of “social distancing” is widely used to slow the spread and transmission of coronaviruses. In many countries, government-imposed lockdowns have lasted for months, forcing many economic sectors to shut down. Many family firms, such as manufacturing companies, restaurants, hotels, gyms, movie theaters, brick-and-mortar retailers, and travel-related companies have been closed by public mandate or forced to drastically reduce capacity. Some export-oriented manufacturing enterprises that rely on foreign markets are facing challenges such as reduced market orders, increased raw material costs, enterprise shutdowns, and increased operating costs. This has led to a loss of clients, a sharp drop in revenue, and a potentially permanent depletion of talented employees. Supply chains are also under pressure due to production disruptions, material shortages, and border closures. All these challenges strain the managerial, financial, and physical resources of the business. This creates dynamics and uncertainty for the growth of family firms. Family firms are highly vulnerable to shocks due to their limited financial capital and resources, small size, and single product type. Many family firms, especially technology-based ones, have experienced a significant decline in orders and a sharp contraction in the market. How to effectively manage the uncertainty associated with rapid changes in the external environment and ensure sustainable business development has become a practical problem that needs to be solved by family firms.

When internal and external contexts are uncertain and dynamic, and the competitive landscape and technology standards are constantly changing, companies must change their resources and routines to survive, innovate, and achieve sustainable growth [4]. In order to create value and obtain sustainable growth, family businesses must adopt effective and innovative behaviors that both manage their resources and integrate these resources into their operations. Strategic entrepreneurship is an organizational innovation that simultaneously carries out opportunity-seeking and advantage-seeking activities [5]. This synchronization not only allows the company to find new opportunities for building future advantages but also allows the company to create wealth based on its current advantages, which is an important organizational tool for managing uncertainty in the company [6]. Therefore, in a changing and competitive environment, family firms can engage in strategic entrepreneurial actions aimed at maintaining competitive advantage and sustainable development.

In the research stream of family firm strategic entrepreneurship, scholars have made excellent studies on family firms in terms of resource orchestration [7], inter-generational succession [8], family involvement [9], internationalization strategy [10,11], and so on. Related studies have mainly focused on the relationship between strategic entrepreneurial behavior and family member involvement [12], the intergenerational transmission of strategic entrepreneurship in family firms [13,14], The relationship between strategic entrepreneurial activity and family firm growth [10,11], and the exploitation and exploration activity in the family firms strategic entrepreneurship [15,16]. However, a review of the relevant literature finds that there are few studies on the triggering mechanism of strategic entrepreneurial activities in family firms, especially the understanding of “why” and “how” family enterprises choose specific strategic entrepreneurial activities and behaviors. This research gap may make it difficult for existing theories to provide clear and effective action guidance for the strategic entrepreneurial activities of family firms, and eventually lead to the practical problem that managers do not know how to carry out strategic entrepreneurial activities.

In order to address the research gap, this paper takes M Electronics Co., LTD (“M Electronics” for short) as a sample case, which is a technology-based family firm that has maintained sustainable development through strategic entrepreneurial activities in this dynamic and uncertain context. We designed an exploratory case study based on the grounded theory approach. Since its establishment, M Electronics has experienced two major strategic changes. In each period, M Electronics has been able to effectively address challenges and achieve sustainable growth through appropriate strategic entrepreneurial activities and behaviors. Therefore, the research objective of this paper is to explore how family firms can effectively cope with external uncertainty risks, enhance employee well-being, and obtain sustainable growth through positive strategic entrepreneurial behavior in a dynamic context. We wish to obtain some valuable information and discover the general rules through the profound study of the strategic entrepreneurship process within M Electronics, which will provide reference to more family firms seeking to adopt strategic entrepreneurship. To accomplish this research objective, in this study we ask the following questions:

- How does this family firm implement effective strategic entrepreneurial activities at specific stages during the whole entrepreneurial process?
- How does the entrepreneur of the sample family firm always choose the appropriate strategic entrepreneurial behaviors and activities at a specific stage?

It is very interesting and novel to explore and answer these two questions. First, it helps to clarify the micro-driven mechanism of strategic entrepreneurial behavior of family firms and provides new insights for family firms to achieve sustainable development through implementing strategic entrepreneurship. Secondly, it helps to bring into play the initiative of entrepreneurs and provide a reference for decision-making to achieve rapid growth of family firms by choosing appropriate strategic entrepreneurial behaviors. 

This study may offer several contributions. First, this study provides a new explanatory framework for the micro-driven mechanism of entrepreneurial behaviors and activities in family firms. Based on the role and social identity theory of entrepreneurs [17,18,19,20] this study constructs the micro-driven mechanism of strategic entrepreneurial behavior for technology-based family businesses from the perspective of how the entrepreneur’s multiple identities evolve dynamically. The micro-driven and behavioral mechanisms of strategic entrepreneurial activity choices of technology-based family firms in different entrepreneurial contexts are described, providing a theoretical framework for future empirical research and theory development. Second, it enriches the literature on entrepreneurial identity. This study responds to the call of scholars to study the multiple interaction and dynamic evolution of entrepreneur identity [18,21,22,23,24,25]. It is found that entrepreneurial role identities and social identities evolve dynamically with changes in firm development contexts. This study explores the mechanisms by which the multiplicity and dynamism of entrepreneurial identities affect strategic entrepreneurial behavior, which expands the relevant research in the entrepreneurial identity literature and also provides a useful exploration of the role of entrepreneurial subjective initiative in strategic entrepreneurial behavior. Third, this study enriches the theoretical research related to family firms. This paper analyzes the impact of the interactions between an entrepreneur’s multiple identities on resource orchestration, value chain management, as well as opportunity seeking and advantage seeking for strategic entrepreneurship in technology-based family firms. By doing so, it reveals the selection mechanism of strategic entrepreneurial behavior of family firms, which provides a useful reference for wealth creation by, and the sustainable development, of firms under the challenges of complex dynamic situations.

The rest of the paper is structured as follows. Section 2 is the literature review. Section 3 is materials and methods. Section 4 is data coding and robustness analysis. Section 5 reports the research results. Section 6 is the research conclusion. Section 7 is the discussion, in which we present the theoretical contribution and management implications, and point out the research deficiencies for future research.

## 2. Literature Review

### 2.1. Entrepreneur Identity Theory

Entrepreneurs are the main agents of entrepreneurial activity, starting with the formation of an intention to create a new business through the process of identifying entrepreneurial opportunities and selecting the type of business. They are business operators, senior managers, and other actors of economic activity who have an entrepreneurial spirit and show a strong desire to innovate, take risks, and promote reform and development behavior [22]. The entrepreneurial journey gives entrepreneurs the freedom to pursue and achieve their dreams and goals in the process of creating and developing their businesses [17]. By unifying “who I want to be” and “what I can do”, entrepreneurs can realize their personal value. Therefore, entrepreneurial activities are largely regarded as an expression of the entrepreneur’s personal identity [1,26], The construction and management of the entrepreneurial identity are of practical importance in the entrepreneurial journey.

Entrepreneur identity is a set of claims about the founders, organizations, and market opportunities around an entrepreneurial entity that give meaning to questions such as “what we do” and “who we are” [24,25]. Entrepreneur identity is also a series of distinctive identities embodied by entrepreneurs in entrepreneurial activities [18], representing the psychological desire of individuals to become entrepreneurs, and is the cognitive presentation of individuals’ enduring goals, desires, and motivations [26], which will affect the decisions and actions of entrepreneurs at special stages of entrepreneurship. These identities include both what role identity theory calls “the roles that individuals play as entrepreneurs and the expectations associated with those roles” and what social identity theory calls “the self-positioning and categorization of entrepreneurs as members of social groups” [17,18,21,26,27].

#### 2.1.1. Entrepreneur Role Identity

Role identity is based on the behavioral expectations that society holds for individuals, and different individuals internalize these expectations in different ways as they acquire their roles [28]. The theory of role identity focuses on the behavior of individuals related to roles and emphasizes that individuals constitute self-identity by playing the roles of types [26]. The research on role identity in entrepreneurship mainly focuses on the various potential roles that entrepreneurs have in their own businesses [23]. Individuals will have multiple roles, and the salience of each role will vary at different times [20]. People will devote more energy to the more salient role and engage in behaviors and activities related to that role. Entrepreneurs may adopt different roles during specific stages [28]. Entrepreneurs dynamic adjust the salience of different role identities in the identity structure according to the dynamics of business development and will think and act based on this identity [21].

Entrepreneurs would prefer specific roles that are important to their identity in the process of entrepreneurship and put more passion and effort into entrepreneurial activities that conform to the role orientation [20,28]. Three different types of entrepreneurial role identities have been developed based on entrepreneurial passion—inventor, founder, and developer—each of which has distinct characteristics and is associated with different entrepreneurial behavior. Entrepreneurs with the inventor identity are keen on activities related to identifying inventions, seeking new opportunities, R&D new products, and expanding markets. Entrepreneurs who are founders are interested in activities related to business creation and opportunity exploitation, including raising capital and building entrepreneurial teams. Entrepreneurs who are developers are more likely to apply their entrepreneurial passion and efforts to the development of new products and services, new markets, and other activities related to business development and growth, such as attracting new customers and co-creating value [20,23]. Entrepreneurs would prefer specific roles that are important to their identity in the process of entrepreneurship and put more passion and effort into entrepreneurial activities that conform to the role orientation [20,21,23].

#### 2.1.2. Entrepreneur Social Identity

Social identity theory focuses more on the relationship between individuals and groups or intergroups and explains how the behavior of individuals and groups emerges through self-categorization and social comparison processes [29,30,31]. Studies of social identity theory in entrepreneurship have shown that the types of social identities and identity structures that are salient to entrepreneurs over time determine a range of strategic responses to adversity in that business [18,31]. Therefore, this theory also explains how entrepreneurs adopt different behaviors when faced with similar situations [17] and analyzes why some individuals are more likely to relate to certain groups than others [21]. Fauchart and Gruber [17] classified entrepreneurial social identities as Darwinian, Communitarian, and Missionary based on entrepreneurial motivation, self-evaluation, and reference frame. These three identity types encompass different degrees of social inclusivity, extending from the economic interests of the “self” to the community interests of “our” to the overall interests of the industry or society [17,32]. The social motivation of entrepreneurs with a Darwinian identity is to accumulate personal wealth through the creation of for-profit enterprises. They will be keen on competition among peers and will regard the profitability of the enterprise as the main reference for the success or failure of the venture. Entrepreneurs with a Communitarian identity are committed to providing valuable and innovative products and services to community members and look forward to receiving the necessary support from the community. Entrepreneurs with a Missionary identity believe that their companies can be enablers of industry ecosystem improvement and social progress. They are committed to making a positive impact on the well-being of others and to acting in a responsible, transparent, and prudent manner [17,18,20,23].

#### 2.1.3. The Multiple Identities of the Entrepreneur

Usually, entrepreneurial identity structures contain both role identities and social identities, and they have a blend of social and role identities [18]. Both identity types suggest that entrepreneurs can have multiple identities at the same time, but attach variable salience to each identity in the identity structure at different stages and contexts [1,33,34,35]. Additionally, the interaction and dynamic evolution of these identities with different significances motivate entrepreneurs to engage in certain activities and interact with people in a way that confirms their roles [17,20,33].

Social identities influence the value orientation of entrepreneurs, reflecting their social motivations and behavioral expectations. Fauchart and Gruber [17] divide the social identity of entrepreneurs into three types, which are reflected in entrepreneurial behavior and affect the key decisions of entrepreneurial enterprises. Role identity provides a guarantee for the implementation of social identity and is a specific expression of social identity [21]. Entrepreneurs need to construct and constantly evolve role identities that can express social identity [18,23,31]. In reality, the type of identity required by entrepreneurs will change with the growth of enterprises, so the evolution of entrepreneurs’ identity and role transformation are important mechanisms and guarantees for the rapid development of enterprises [34].

However, in this dynamic transformation, there is often a mismatch between role and social identity. In addition, compared with the leaders of mature enterprises, many entrepreneurs are “do-it-allers”. In addition to the role of founder, investor, inventor, developer, etc., they assume the leadership of marketing, operation, finance, and HR. That is, entrepreneurs may sometimes have to deal with a set of simultaneously and sometimes conflicting behavioral expectations [34,35]. Our study aims to show how entrepreneurs adopt several, sometimes overlapping identities, how they evolve in the business growth stages, and what impact this evolution has on the strategic decisions and entrepreneurial behavior of companies.

### 2.2. Strategic Entrepreneurship and Strategic Entrepreneurship in Family Firm

Strategic entrepreneurship is the integration of entrepreneurial behavior and strategic activities in the formulation and adoption of actions aimed at addressing challenges and creating wealth by companies [5,6,36]. Strategy is made up of decisions, reactions, and activities that increase the chance for success and decrease the risk of failing to meet the goals [37]. The fundamental question of strategic management is how organizations achieve and maintain their competitiveness. Entrepreneurship is the process of identifying and capturing opportunities and thereby creating novel products and services and realizing their potential value. Therefore, strategic entrepreneurial behavior allows companies to maintain their current competitive advantage through strategic behaviors to obtain high profits, and also to explore new opportunities through entrepreneurial behavior to achieve sustainable growth [15,38]. Therefore, opportunity-seeking and advantage-seeking are the two main activities of strategic entrepreneurship [36,38]. This concept was first proposed in 2001 [5] and has been in development for 20 years now.

Early research described strategic entrepreneurship from a process perspective as the successful combination of strategic management and entrepreneurial activities, including strategic behaviors taken from an entrepreneurial perspective and entrepreneurial behaviors taken from a strategic perspective, as well as generating profits and obtaining sustainable growth for the firm [36,37].

In recent years, many scholars have conducted continuous research on strategic entrepreneurship and have yielded numerous research achievements in terms of dimensions and elements [5,36], entrepreneurial and market-oriented [36], and exploration activity and exploitation activity [15]. Specifically, Hitt et al. [5] pointed out strategic entrepreneurship is mainly composed of two behaviors: entrepreneurial opportunity-seeking (exploration behaviors) and strategic advantage-seeking (exploitation behaviors). Schindehutte et al. [39] argue that strategic entrepreneurship is an independent paradigm, a conceptual field of how decision-makers can exploit the creative potential of complex dynamic contexts. Simsek et al. [40] emphasize that strategic entrepreneurship is a logical system of behavioral decision-making for firms. Although scholars have described strategic entrepreneurship differently, which may lead to a blurring of the boundaries of strategic entrepreneurship, there is a basic consensus that strategic entrepreneurship is an organizational innovation that integrates two behaviors of opportunity-seeking and advantage-seeking [38,41,42].

The sustainable growth and value creation of a firm is not only a process of constantly seeking opportunities but also a process of seeking strategic advantages through resource orchestration. Therefore, strategic entrepreneurship theory organically combines the value-creating behavior and advantage-building behavior of firms and provides a new perspective for the sustainable growth of a firm [43]. Through the integration of entrepreneurial and strategic behavior, strategic entrepreneurship can use entrepreneurial behavior to identify and develop entrepreneurial opportunities, so that enterprises can open new markets or provide new products and services, and ultimately achieve the purpose of seeking greater value creation. In addition, strategic behavior can help explore key resources and resource allocation for enterprises to establish competitive advantages in an uncertain dynamic environment to realize the organic combination of enterprise value construction and competitive advantage building [37,44].

The study of strategic entrepreneurship in family firms has received more academic attention since 2011 and has accumulated certain academic outputs after more than 10 years of research. Related studies have focused on resource orchestration and the implementation of strategic entrepreneurship [7], intergenerational transmission of family firms and sustainability of strategic entrepreneurial activities [13], family-member involvement and entrepreneurial strategy decision-making [10,12], and so on. However, existing studies lack research on the micro-behavioral mechanisms of strategic entrepreneurship of family business entrepreneurs at different stages of business development. This leaves the strategic entrepreneurial activities of the firm potentially lacking explicit and effective behavioral guidance, and entrepreneurs do not know how to implement strategic entrepreneurial behaviors to effectively respond to the challenges of dynamic situations. The purpose of this study is to deal with this theoretical gap and to explore how and why family enterprises choose different strategic entrepreneurial behaviors to effectively reduce risks and achieve sustainable growth of enterprises through an exploratory case study.

### 2.3. Research Review

The literature review found that the content and perspectives of research on strategic entrepreneurship in family firms are diverse and interdisciplinary. Researchers have analyzed the connotations, functions, and outcomes of strategic entrepreneurship from multiple perspectives, but not much research has been conducted on the micro-driving mechanisms of strategic entrepreneurial behavior. At the same time, although scholars recognize that entrepreneurial identity has important implications for corporate strategy and behavior, there is a dearth of research on how entrepreneurial identity affects corporate entrepreneurial behavior and how the multiplicity and dynamics of identity relate to corporate strategic entrepreneurial behavior. In particular, how entrepreneurs construct and dynamically evolve their entrepreneurial identities and the impact of these identity structures on entrepreneurial behavior has yet to be studied in depth [44].

## 3. Materials and Methods

### 3.1. Method Selection

In accordance with the purpose of the study, a longitudinal single case study was designed for theoretical construction. The data analysis process follows grounded theory methods. The reasons for this choice can be listed as follows. 

First, we sought to understand “why” and “how” family firm entrepreneurs choose specific strategic entrepreneurial behaviors in the process of sustainable development. Given the limited theories on this issue, we hope to generalize the relevant theories from the selected case companies by conducting an in-depth study. The case study approach is suitable for explaining research issues such as explanation mechanisms or processes [45,46]. Therefore, it is appropriate to choose a case study for this research.

Second, the strategic entrepreneurial activities of family firms are a dynamic and continuous process involving multi-stage interactions of technology, management, family, etc. Longitudinal case studies, on the one hand, help to discover patterns from complex phenomena, uncover the hidden theoretical logic behind them, and facilitate the induction and presentation of interrelationships among multiple constructs; On the other hand, this method supports the presentation of detailed evidence to demonstrate the multiple dimensions and multiple stages of the change process to provide a basis for theory construction [45,47]. In addition, this study requires an in-depth analysis of the entrepreneurial behavior of entrepreneurs in different periods, so a longitudinal case study is chosen to present the process of a representative family firm successfully coping with challenges and obtaining sustainable development through appropriate strategic entrepreneurial behavior in a dynamic situation. Therefore, it is appropriate to choose a longitudinal case study for this study.

Third, given the need for an in-depth analysis of the relationship between the dynamic evolution of entrepreneurs’ multiple identities and strategic entrepreneurial behavior, a single case study is conducive to long-term tracking and in-depth research of research topics, and can more clearly show the mechanism of action between various constructs, so it is appropriate to adopt a single case longitudinal analysis. This is also in line with the principles of representativeness and typicality proposed by Yin [45] in the selection of single-case studies. Therefore, it is appropriate to choose a longitudinal single case study in this study.

Fourth, the grounded theory approach is suitable for exploring patterns of behavior in dynamic contexts in order to derive new theoretical elements from qualitative data [48,49], and for answering the “why” and “how” elements of our research issues [50].

### 3.2. Case Selection

The sample selection mainly considers the matching between the target enterprise and the research problem. According to the research problem, this research sets the following selection principles: First, the industry in which the enterprise is located should belong to the manufacturing industry, and the sample enterprise should have a strong representation in the industry; Second, the sample enterprise should experience a complete stage from start-up and growth to rapid development or expansion, and be able to deal with risk challenges through effective strategic entrepreneurial activities, which can more completely reflect the mechanism of action and specific details of strategic entrepreneurship. This is very important for theory construction because it is ”particularly suitable for illuminating and extending relationships and logics” [46]. 

The research team screened twelve candidate family firms to arrive at M Electronics as the sample firm. The company is mainly engaged in the development and production of nanocrystalline soft magnetic materials and demonstrates behaviors that suggest it can survive and have a good development prospect in the competitive magnetic materials industry. The reasons can be listed as follows:

First, the magnetic material industry provides the best context for the research topic. It is a technology-intensive and highly interrelated industry with upstream and downstream enterprises. Competition among peer firms within the industry is intense around technology segments, which makes it an ideal industry for us to conduct strategic entrepreneurship research on technology-based family firms.

Second, since its inception in 2008, M Electronics has effectively responded to various challenges to obtain rapid growth through aggressive strategic entrepreneurial behavior. In 2016, the entrepreneur achieved expansive growth based on his technological advantages. The enterprise is highly typical and inspiring, suitable as a sample company for in-depth analysis to find out its success code to serve for theory construction.

Thirdly, M Electronics is a family enterprise with technology as its core competitiveness, and the entrepreneur is involved in the entire life cycle of the firm, including creation, growth, and expansion. The entrepreneur dynamically adjusts his identity structures at each stage of the enterprise, making this firm suitable to analyze the strategic entrepreneurial process of the company from the perspective of the evolution of the entrepreneurial identity.

Fourth, a member of the research team had been consulted by the managers of M Electronics, which give us the opportunity to track and pay attention to the development of the company for a long time, which can guarantee the availability and reliability of the research data.

Therefore, it is appropriate to select M Electronics as the subject of this study.

### 3.3. Case Company

#### 3.3.1. M Electronics

M Electronics was established in June 2008 and is located in the Fengxian Industrial Park in Shanghai, China. The owner and founder of M Electronics is Mr. Tao Zhang. M Electronics is a technology-based family firm that focuses on the development, production, and sale of nanocrystalline soft magnetic materials. This firm has a certain scale now after more than 10 years of development. Tao Zhang’s wife, brother-in-law, parents-in-law, and other relatives and friends have been engaged in the management and production of the company since its inception.

The main products of M Electronics include non-crystalline cores, super microcrystalline cores, cobalt-based amorphous cores, etc. The products are widely used in sensors, inverters, electronic components, and electric control systems in the information and communication, electric power, and electronic industries. In recent years, these products have gradually become the core materials for high-grade manufacturing products in aerospace, new energy, electronics and electric power, medical, and other industries. The main raw material of the company is super microcrystalline strips. In 2016, M Electronics achieved scale expansion based on technical advantages and established R Electronics Co., Ltd. (R Electronics).

The main family members working in the company are Zhang’s wife, Zhang’s brother-in-law, and his parents-in-law. His wife is responsible for daily operations, finance, and HR, while Zhang’s brother-in-law and parents-in-law are mainly involved in support services. They all joined the company from the beginning of its establishment in 2008. In addition, we interviewed Mr. Li, the purchasing supervisor, and Mr. Han, the production manager, both of whom joined the company in 2009; Ms. Wang, the warehouse supervisor, who joined in 2013; and Mr. Liu, the technical director, who joined in 2011, during the on-site conversation

#### 3.3.2. M Electronic Development Stage

By analyzing the qualitative material of M Electronics, we found that the development process of the company showed three main distinct phases.

After graduating from university in 2001, Tao Zhang engaged in R&D for nearly seven years in a technology company. During this period, he mastered the technology of the R&D and production of nanocrystalline soft magnetic materials, which laid the foundation for his later venture.

Initial stage (2008–2011)

In 2008, Tao Zhang resigned from his original job and came to Shanghai to launch his business. During this period, he completed his identity transition from an employee to an entrepreneur. The main entrepreneurial activities of the entrepreneur in this stage include creating and running the company, acquiring customers, product development, and market development. the entrepreneur quickly commercializes technological advantages and provides the foundation for product reputation and corporate legitimacy building while gaining customer recognition.

Growth stage (2011–2015)

After its founding and development by Tao Zhang, the business volume and market size of M Electronics grows. During this period, entrepreneurs focus on building good community relationships, meeting the individual needs of community members by continuously developing new products and services, and expecting to obtain the necessary help from the community.

Expansion stage (from 2016 to date)

During this period, M Electronics encountered problems such as the untimely supply of raw materials and unstable quality. This led to problems such as delayed delivery of orders and degradation of product parameters for the company. Tao Zhang actively responded to community concerns and grasped opportunities based on technical advantages, completed the expansion of the enterprise in 2016, established a raw material production enterprise through value chain extension, and focused on the development and production of super microcrystalline strips, which effectively alleviated the quality problems faced by M Electronics in raw material procurement. Since 2020, the company has added three new production lines, which effectively meet the growing market demand, and the products continue to be recognized by customers.

### 3.4. Data Collection and Data Processing

#### 3.4.1. Data Collection

The data collection strategy needs to match the research questions. For this study, the issues concerning the evolution of entrepreneurial identity and the implementation of strategic entrepreneurial activities are relatively subjective behavioral procedures, and relevant qualitative material can be obtained through a variety of sources. The research team has been tracking M Electronics for nearly three years, selecting appropriate data collection strategies to form data triangulation validation. Throughout this process, we maintained a cautious, neutral, and unbiased attitude to minimize the impact on the interviewees as much as possible.

First, we collected data through different information sources, which is important for forming complementary perspectives and reducing informant bias in the case analysis. When selecting interviewees, we interviewed production, procurement, technology, and other departments, involving managers at different levels to obtain different perspectives on the implementation process of strategic entrepreneurial behavior in the company, which ensures triangulated validation of different types of data. 

Second, we combined a variety of data collection methods, including semi-structured in-depth interviews, on-site observations, informal interviews, and secondary sources, among which, semi-structured interviews and on-site observations were the main data sources. From March 2020 to September 2021, the research team conducted seven surveys of M Electronics, involving 19 managers and employees, and 26 interviews; at the same time, the team surveyed one supplier and three customers with whom they worked many times. Each interview was recorded and audio-recorded and was organized as soon as possible after the interview, resulting in a 500,000-word transcript (See Table 1). 

Interviews

In an unstructured interview, we ask the interviewee to talk about “some stories about your company”, a process in which we mainly listen and try not to interrupt the interviewee. If new clues emerged from listening, follow-up questions could be asked at the end of the interviewee’s narrative. The formal interviews lasted between one and two hours, during which members of the research team conducted semi-structured interviews with entrepreneurs, family members, corporate executives, and employees.

Next, we conducted ongoing interviews on emerging theoretical themes. For example, we asked the entrepreneur to talk about any difficulties they faced during the entrepreneurial process and about how they addressed those difficulties. In the vast majority of cases, many of the relevant questions in this area were already answered when telling their company story. Once we discovered the importance of “entrepreneurial identity” to our theory construction, we conducted deeper data collection and in-depth interviews on this subject. 

After each interview, we recorded and transcribed the interview materials as quickly as possible, taking note of any ambiguities and inconsistencies for the next supplementary interview. This was finally transcribed into approximately 367 single-spaced pages. The average interview length was 79 min, with a range of 53 min to 5 h.

On-site observations and conversation

We supplemented the interview material with on-site observations, including plant visits and facility tours, which allowed us to observe the interactions between employees and various stakeholders and entrepreneurs without interruption. During the face-to-face conversation sessions, interviewees were asked to describe their role in the business, followed by their attitudes toward the entrepreneur and the business. During this process, the researchers explored additional details and stories around what seemed to be important to the interviewees. 

The documents

To ensure the triangulation of qualitative data, we also obtain secondary data through a number of documents, such as company brochures, publications, financial statements, annual reports, archives, media events, and other documents. In addition to this, we also took a large number of on-site photographs and wrote a large number of field notes that could be used to triangulate the data at a later stage. (See Table 1).

#### 3.4.2. Processing of Qualitative Data

Drawing on the research methodology and data processing process of the exploratory case study [46], the data processing in this study was carried out in the following steps.

First, members of the research team quickly organized the interview data at the end of each interview and reviewed the literature to clarify the construct and research ideas. In addition, any unclear or newly discovered questions were organized for in-depth follow-up in the next interview. 

Second, based on Yin’s [45] research perspective, we use an analytical strategy of matching the identity structure and behavior of the entrepreneur at different time points to study the sustainability model and innovation mechanism of M Electronics.

Third, following the principles of problem orientation, triangulation verification, and avoidance of subjective preferences, the research team members ensured that the distilled conceptual logical relationships were supported by at least two or more sets of qualitative data without prior assumptions or theoretical stereotypes, and the research team members finally reached a unity of opinion and distilled the research results through repeated discussions and screening.

## 4. Data Coding and Robustness Analysis

Data analysis combined methods from the longitudinal case study [45] and grounded-theory methods [47,49] to trace the trajectories followed by the case under study, identify behaviors and activity patterns in those trajectories, and abstract their common patterns. 

### 4.1. Coding 

The data encoding follows three steps: open coding, axis coding, and selective coding [47,49]. This allows the relationships between constructs to automatically emerge and provides support for theory construction.

#### 4.1.1. Open Coding

First, the research team open-coded the original data obtained from the research. Through open coding, the ponderous raw data became progressively clearer and easier to navigate and compare. Open coding is a process of analyzing qualitative data and extracting and characterizing thematic categories. This process follows the principle of openness in theory construction and is as close to the data as possible to form a rich topic of the strategic entrepreneurial process of the firm. We decomposed and organized the discourses and original data obtained from interviews and on-site observations according to the sequence of events, then scanned and refined the relevant constructs in the events, and assigned labels to the constructs. Second, the relevant constructs were clustered into first-order concepts. According to the development stage of the enterprise, the events related to the research topic of this paper were extracted from the original data to form the relevant constructs, and the constructs that appeared more than three times were retained to determine the first-order concepts. Finally, we extracted a total of 96 concepts and 48 first-order concepts. Table 2 shows the representative quotations of open coding.

#### 4.1.2. Axis Coding

The main task of axis coding is to integrate multiple first-order concepts that are logically related in order to obtain second-order themes. Following the logical paradigm of “condition-behavior-outcome”, several first-order concepts are connected, and, based on the logical relationships behind the concepts, the clustered themes are grouped into the same second-order themes. One category at a time is analyzed in depth to probe deeply into the relationships between first-order concepts and second-order themes. The same approach was used to discover and establish associations between second-order themes and aggregated dimensions. For example, constructs such as “ control management cost” and “control labor cost” were conceptualized as the first-order concept of “cost advantage,” and then the first-order concepts of “cost advantage” and “efficiency advantage” were categorized into the second-order theme of “advantage-seeking,” and then “opportunity-seeking“ and “advantage-seeking“ were integrated into the aggregated dimension of “strategic entrepreneurship” to form a complete evidence chain. (see Table 3).

After continuous abstraction, comparison, extraction, and induction, we clustered the 48 first-order concepts obtained from open coding into 24 second-order themes by axis coding and then merged them into seven aggregated dimensions, such as entrepreneur social identity, entrepreneur role identity, entrepreneurial well-being, entrepreneurial passion, strategic entrepreneurship, and so on. The connotation and nature of the partial aggregation dimensions are described as follows.

Entrepreneur social identity. The construct of “entrepreneur social identity” is named with reference to the views of Fauchart and Gruber (2011) [17]. Entrepreneur social identity may help explain why entrepreneurs facing similar contexts may act differently through self-categorization and social comparison. In this case, the social identity of the entrepreneur presents a “Darwinian” identity at the initial stage, a “Communitarian” identity in the entrepreneurial growth stage, and a “Missionary” identity in the expansion stage.

Strategic entrepreneurship. The construct of “strategic entrepreneurship” is named with reference to the views of Ireland and Webb (2009) [41]. Strategic entrepreneurship is a summary of a series of activities in qualitative materials that entrepreneurs seek to upgrade their technological advantages and seek entrepreneurial opportunities through resource integration, value chain management, process optimization, and other activities based on different entrepreneurial situations.

Entrepreneurial well-being. The construct of “entrepreneurial well-being” is named with reference to the views of Wiklund et al. (2019) and Wach et al. (2021) [51,52]. Entrepreneurial well-being is a summary of a series of positive emotions, satisfaction, and positive psychological function. In this case, entrepreneurial well-being mainly includes personal acceptance and self-recognition, personal growth, psychological health, self-sufficiency, and positive relationships.

#### 4.1.3. Selective Coding

**Figure 1:**
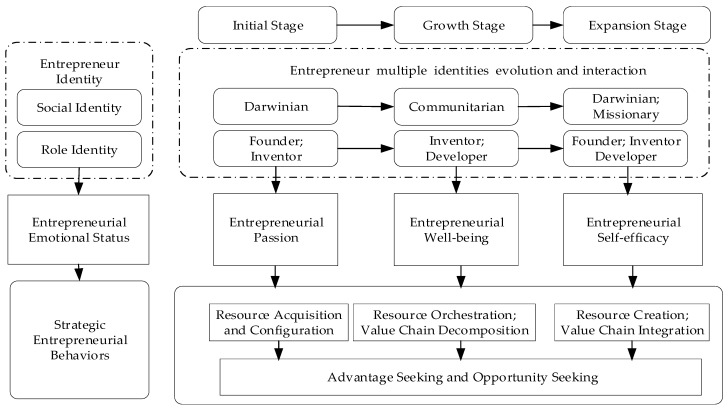
Theoretical Model.

Selective coding aims to analyze the relationships among the aggregated dimensions, condense the core categories, and construct a grounded theory model based on the logical relationships among the core categories. The key activity in this step is to explore a “storyline” that serves as a “hook” from the aggregated dimensions to string all categories into a core category. Based on the characteristics of the sample firms, and through a continuous comparison of the obtained aggregation dimensions with existing theories, we found that the core category of this paper can be formulated as follows: the evolution of the multiple identities of entrepreneurs affects the strategic entrepreneurial behavior of firms in a given period through different entrepreneurial emotional. The “storyline” can be summarized as the “dynamic interaction of multiple entrepreneurial identities—different types of emotions—strategic entrepreneurial behavior”. Figure 1 is the theoretical model.

Figure 1 summarizes the mechanisms of family firm entrepreneurs’ multiple identity interactions on strategic entrepreneurial behavior. The theoretical model is a very important and interesting tool to make us more intuitive about this microscopic influence mechanism. The identity types in the entrepreneurial identity structure will evolve and interact with each other, and this dynamic interaction will affect the different emotional states of entrepreneurs and make them choose different behaviors to promote the implementation of strategic entrepreneurial activities. In the results section, we will elaborate on Figure 1 based on the different stages of business development.

### 4.2. Saturation Test

The entire study process of collecting and analyzing the data took nearly two years. Qualitative data collected from different sources were cross-validated using triangulation methods to improve the reliability and stability of the data. In addition, the data were continuously supplemented, checked, and verified through supplementary interviews, and timely additional communication with the entrepreneur of the sample company on unclear or ambiguous topics was conducted several times. The data analysis reports were repeatedly sought for review by the relevant interviewees of the company. According to the feedback, we made appropriate adjustments and improvements to the relevant data. Finally, to ensure theoretical saturation, we tested again with the remaining one-third of the interview transcripts, and no new categories or relationships were found. Therefore, it can be considered that the core clustering and theoretical model of this case have reached saturation.

## 5. Results

The entrepreneurial journey gives entrepreneurs the freedom to pursue and achieve their dreams and goals in the process of creating and developing their businesses [17]. By unifying “who I want to be” and “what I can do”, entrepreneurs can realize their personal value. Therefore, entrepreneurial activities are largely regarded as an expression of the entrepreneur’s personal identity [1,26], and entrepreneurs will construct and manage their own identities to obtain entrepreneurial identities that meet the requirements of enterprise development [23,43,53].

Through qualitative data coding, it can be found that, at different stages of the enterprise, the entrepreneur flexibly and dynamically adjusts his social identity and role identity types according to changes in the development environment of the enterprise, and integrate and reconstruct internal and external resources to effectively promote the implementation of strategic entrepreneurial behaviors to enable companies to meet the challenges and maintain the momentum of sustainable growth.

### 5.1. Initial Stage of Entrepreneurship

#### 5.1.1. The Multiple Identity Types of the Entrepreneur in the Initial Stage

In this stage, the multiple identity types of entrepreneurs are Founder, Inventor role identity, and Darwinian social identity.

According to the survey data coding, the entrepreneur worked in a high-tech enterprise in Beijing for nearly 7 years after graduation. During this period, he was paid an average salary and could not bring his family to live together in Beijing, so his motivation was to use his technological advantages to create enterprises and accumulate personal wealth in order to improve the quality of life of their families. Therefore, the type of social identity of entrepreneurs at this stage is mainly Darwinian. In order to create a profitable business as quickly as possible, the entrepreneur needs to adjust their role identity accordingly.

*“My idea at that time was to make more money and improve the living conditions of my family through the technology I mastered.” (Interview with entrepreneur Tao Zhang)*

On the one hand, he needs to clarify his role identity as a founder. He should strive to raise funds and build teams, and get resources from his family and friends. On the other hand, based on previous experience, the entrepreneur obtains the information resources needed to start a business by scanning and searching for market and technology information. Meanwhile, the entrepreneur uses his inventor role identity to integrate the acquired information with his technical advantages and develop and produce competitive products to quickly obtain profits by quickly completing the transformation of technical advantages into applications.

*“In 2008, the government issued policies to support entrepreneurship. After discussing with my family, I resigned decisively and came to Shanghai to start my own business.” (Interview with entrepreneur Tao Zhang)*

Therefore, in this stage, the multiple identity types of the entrepreneur are a Darwinian social identity and the role identities of a founder and inventor.

#### 5.1.2. Multiple Identities Interaction and Entrepreneurial Passion

Entrepreneurial passion is a strong and positive emotional experience of entrepreneurial activity held by entrepreneurs. People with high entrepreneurial passion are able to identify with some meaningful and salient identities and will put more time and effort into entrepreneurial activities related to those identities [20]. Entrepreneurial passion enables entrepreneurs to devote more energy and effort to adapt and cope with entrepreneurial challenges [20]. Moreover, when an entrepreneur’s multiple identity types can support their entrepreneurial motivation, it will effectively enhance their entrepreneurial passion and make them have more impulse and willingness to engage in and experience these entrepreneurial activities [28].

In the initial stage, the entrepreneur’s role identities and social identities are consistent and mutually supportive. A Darwinian social identity provides effective social motivation and action guidelines for entrepreneurial behavior, while founder and inventor role identities give behavioral support for this motivation. The consistency and interaction between social identity and role identity can enhance entrepreneurs’ entrepreneurial passion. First, when the entrepreneur’s social motivation and behavioral support are highly consistent, i.e., when the “*what I want to do*” embodied in the entrepreneur’s social identity is consistent with the “*what I can do*” reflected in the role identity, it will make the entrepreneur believe that he can engage in meaningful things. Secondly, when an entrepreneur engages in entrepreneurial activities that they “*want to do and can do*”, they will be willing to invest more passion and effort in entrepreneurial activities when they can not only meet the needs of self-value realization but also improve the living conditions of their families through entrepreneurial activities. Therefore, the entrepreneur is full of high entrepreneurial passion for entrepreneurial activities in this stage.

*“When I have a business plan, I pay special attention to the information about raw material procurement, customers, market dynamics, etc. I usually spend more time to understand the entire production and processing process of the product.” (Interview with entrepreneur Tao Zhang)*

#### 5.1.3. Multiple Identity Interaction, Entrepreneurial Passion, and Strategic Entrepreneurship

The interaction of multiple identities in the initial stage improves entrepreneurial passion, which has a positive effect on entrepreneurial perception, entrepreneurial behavior, opportunity identification, and the technology-commercialization speed of entrepreneurs. Meanwhile, the initiative of the entrepreneur will gain positive perception and resource support from employees and investors. Entrepreneurial passion will also encourage the entrepreneur to use more innovative ways to piece together internal and external resources based on technological advantages, which can accelerate the R&D and production of initial products, and promote the rapid commercialization of technological advantages and obtain profits to bring more opportunities and advantages for the enterprise. 

Opportunity- and advantage-seeking are two important activities of strategic entrepreneurship. Next, we discuss the impact of multiple identity interactions and entrepreneurial passion on strategic entrepreneurship from these two aspects.

Opportunity-seeking. Entrepreneurial passion encourages the entrepreneur to spend more time and energy on information acquisition, knowledge learning, and other activities. Based on his role identity of inventor, the entrepreneur will integrate the knowledge and information acquired with the technological advantages to improve the ability of new product R&D. Meanwhile, entrepreneurial passion also stimulates the entrepreneur to innovate cooperation models based on his founder identity to explore various potential market opportunities and achieve new customer acquisition.

*“I promised this customer about one cycle after the payment of the goods. After I got the deposit I grabbed the materials and carried out tests and production according to the product parameters required. After the first batch qualified on testing, I got the second order …… We delivered the goods every two or three days. Eventually, this customer became our core customer now.” (Interview with entrepreneur Tao Zhang)*

Advantage-seeking. According to the emotional contagion theory, the entrepreneurial passion of a family business entrepreneur is also contagious. The positive and strong feelings of the entrepreneur will be transmitted to their family, employees, and investors through social comparison and emotional imitation mechanisms [54] to obtain the identification of stakeholders. During this period, the technological strengths and entrepreneurial enthusiasm of entrepreneurs can raise employees’ optimistic expectations about the company’s prospects, resulting in greater organizational commitment and work engagement. This will not only enable entrepreneurs to obtain emotional encouragement from stakeholders but also obtain their necessary support and willingness to cooperate.

Firstly, the technological advantages and entrepreneurial passion of the entrepreneur can attract family members, relatives, and friends to work in the enterprise, which to a large extent reduced the labor cost in this stage.

*“My wife, brother-in-law, parents-in-law, and Manager Han followed me from the beginning to ensure the stability of the core team and effectively reduced the labor cost in the early stage of the business.” (* *Interview with entrepreneur Tao Zhang)*

Moreover, family members and friends also provide financial support and emotional encouragement to the entrepreneur. They also actively provide the necessary information and other support for the entrepreneur through various social relationships, so that the entrepreneur obtains the advantage of resource costs.

*“Now think about it, at that time, without the encouragement of family and friends, without the support of various resources provided by them, I’m sure I wouldn’t have stuck with it, let alone have it today.” (Interview with entrepreneur Tao Zhang)*

Secondly, Zhang Tao’s entrepreneurial passion and entrepreneurial persistence enable stakeholders to have a deeper perception of him. They have more optimistic expectations about the consistency between the identity type of entrepreneur and his entrepreneurial activities and are willing to provide support for their product R&D. In particular, the entrepreneur can also obtain more employee commitments. The employees will actively cooperate with the entrepreneur to carry out innovation experiments. This will accelerate the commercialization of technological advantages, and finally enhance the competitiveness of enterprises.

*“At that time, to get an order, I promised the customer not to charge R&D fees and mold fees. If the sample trial qualified, and then talk about cooperation. The customer agreed based on his understanding of me After receiving the order, the staff actively cooperated with the test, and the sample soon met the customer’s requirements. In the subsequent new product research and development, the technicians of both sides cooperated well, which greatly shortened the research and development cycle. “ (Interview with entrepreneur Tao Zhang’ wife)*

An example of the data structure in the initial stage is shown in Table 4. Therefore, we proposed:

**Proposition** **1.** *In the initial stage, the interaction between the entrepreneur’s Darwinian identity and their Founder identity enhances the entrepreneurial passion and motivates the entrepreneur to complete the implementation of strategic entrepreneurial activities through behaviors such as resource acquisition and integration.*

### 5.2. Growth Stage of Entrepreneurship

#### 5.2.1. The Multiple Identity Types of Entrepreneurs in the Growth Stage

The multiple identity structure of the entrepreneur in this period is a Communitarian social identity and inventor and developer role identities.

By 2011, the entrepreneur had established increasingly stable interest ties with suppliers, customers, and peers. The company and community members are increasingly cooperating with each other, and gradually forming a collaborative and symbiotic community relationship with each other. The cooperation between them has been strengthened, and the external network of the enterprise has been expanded. The main activities of the entrepreneur have gradually changed from the creation of profitable enterprises at the initial stage of entrepreneurship and the acquisition of market legitimacy to the maintenance of customer relations. They hope to get more order shares through the improvement of community relations and give full play to their technical advantages to strive to provide more valuable products and services for community members. Therefore, the entrepreneur’s social identity gradually evolves from a Darwinian to a communitarian identity. In addition, in order to better meet the needs of community members for personalized products, the entrepreneur’s role identity has evolved from the founder who focused on enterprise creation in the initial stage to the developer role identity. The entrepreneur needs to develop new products and services through the absorption and utilization of knowledge.

*“Normally, I am also very concerned about the absorption and utilization of some new technologies and new processes, and we (community members) often discuss among ourselves, and some new products are developed, and they are willing to try them out and send back timely feedback to facilitate our improvement. What new needs they have, I also play their own technical advantages, and do their best to meet.” (Interview with entrepreneur Tao Zhang)*

Therefore, the multiple identity structure of the entrepreneur in this period is a Communitarian social identity, and inventor and developer role identities.

#### 5.2.2. Multiple Identity Interaction and Entrepreneurial Well-Being in the Growth Stage

Entrepreneurial well-being is defined as “the emotional experiences associated with positive emotions, satisfaction, and positive psychological function related to the construction, maintenance, growth, and operation of entrepreneurial firms”. Entrepreneurial well-being mainly consists of personal acceptance and self-recognition, personal growth, psychological health, self-sufficiency, and positive relationships [51,52]. 

In the growth stage, with the business becoming more efficient, the living conditions of the entrepreneur and his family are improved. In addition, with the improvement in company regulations and functional departments, the entrepreneur can also be relieved of the tedious routine management and can devote more energy and effort to the technical area. “Expectancy theory” suggests that the difference between expectations and actual achievement is related to subjective well-being [55]. In this stage, the entrepreneur can carry out innovative experiments such as technological progress and product optimization based on his role identity as a developer and inventor in the technology field. These activities help the entrepreneur to provide community members with higher quality products and meaningful services based on a Communitarian identity, and gain strong identification and knowledge sharing from community members.

*“Now our factory environment is much better than before, our family’s living condition is also much better before, and our children are now coming here to study. “ (Interview with entrepreneur Tao Zhang)*

*“Daily management is her (entrepreneur’s wife) to do, like procurement, order decomposition, customer maintenance, shipping and so on I do not worry about, to be able to do something they want to do, I usually care more about the heating furnace and magnetization furnace these links process improvement.“ (Interview with entrepreneur Tao Zhang)*

Entrepreneurs will integrate technological advantages and acquired knowledge, further improve product profits and market competitiveness, and finally achieve a relatively satisfactory positive emotional experience of life and entrepreneurial activities, and improve entrepreneurial happiness. The entrepreneur integrates technical advantages and acquired knowledge to significantly increase product profitability and market competitiveness. These experiences bring positive emotional experiences to the entrepreneur’s state of life and entrepreneurial activities and ultimately enhance his entrepreneurial well-being.

*“(Customers) still recognize our R&D capability and products, sometimes just individual spot checks. They are also the first to give us feedback when they have ideas about product improvements or new needs, so that we can help improve and develop them.” (Interview with entrepreneur Zhang’s wife)*

#### 5.2.3. Multiple Identity, Entrepreneurial Well-Being, and Strategic Entrepreneurship

Communities with high entrepreneurial well-being have a freer and more creative and inspiring environment for innovation [56]. For the entrepreneur of a technology-based family firm, his Communitarian identity will not only make him inclined to satisfy the existing needs of the community members but also to take the initiative to develop new market needs to provide highly personalized products for community members and realize benefit sharing. Meanwhile, the entrepreneur’s inventor role identity will help him to search for new opportunities and discover new needs in his community market; and his developer role identity will make him focus more on obtaining information about new product feedback and market dynamics. The acquisition of such information can facilitate the entrepreneur to objectively evaluate his abilities and set realistic goals, and also facilitate the entrepreneur to overcome the interference of environmental changes under the situation of rapid market changes, fiercely competitive environment, and rapid technological iterations to focus more energy on his own strengths and areas of expertise to carry out technological innovation activities, so that he can realize his own value by improving business efficiency and obtain new and more entrepreneurial happiness. This is a virtuous cycle.

In addition, entrepreneurial well-being induces entrepreneurs to maintain entrepreneurial behavior and influences their entrepreneurial initiative [57], and entrepreneurial well-being is also an energetic source for entrepreneurial self-regulation [52], which enables entrepreneurs to take initiative and invest enough energy to identify problems and seek solutions to them.

*There was a period of time when the company needed to stagger production and work overtime due to the increased variety and volume of orders, which overlapped with Shanghai’s strict environmental regulations and production restrictions for major events, creating challenges to production continuity and on-time, which led to many employee complaints and frustrated work ethic. Then some employees suggested that we should optimize when taking orders, and some orders are not only small in volume but also troublesome to produce, they suggested that we should not take such orders in the future.” (Interview with entrepreneur Tao Zhang)*

Based on prudence and comprehensive considerations, the entrepreneur made the decision to horizontally decompose the value chain based on employees’ opinions. Horizontal decomposition of the value chain is a value management model in which a firm transfers non-core businesses in the firm’s value chain to other firms through licensing, outsourcing, or transfer [58]. The company seeks to purchase third-party services for some non-core businesses such as logistics, auditing, advertising, and certification, seeks peer-to-peer substitution for some low-margin and niche orders, and focuses the company’s superior resources on the process by which it can leverage its technological advantages.

For M Electronics, the entrepreneur achieves corporate governance through a fusion of ownership and control, which has a unique economic and non-economic value. For example, the informal governance structure formed by the entrepreneur relying on his own technical advantages and a package of interpersonal relationships, and the hybrid governance mechanism relying on ownership, authority, interpersonal trust, and non-institutional control, can not only fully release the entrepreneurial spirit and help himself and his employees to obtain entrepreneurial happiness but also has the advantages of quick decision-making and flexibility, which is conducive to the company to focus on superior resources and domains, grasp innovation opportunities, and achieve the purpose of opportunity seeking and advantage seeking.

Next, we will explore the impact of the entrepreneur’s multiple identity interactions and entrepreneurial well-being on the opportunity-seeking and advantage-seeking activities of strategic entrepreneurship during the growth period.

Opportunity-seeking. The interaction of role identity and social identity enables entrepreneurs to pursue entrepreneurial well-being, and this emotional experience can be effectively enhanced by conducting innovative experiments in areas that he is interested in and good at. At the same time, with the increase in business, entrepreneurs divest non-core businesses and focus on core businesses through value chain horizontal decomposition.

Value chain horizontal decomposition has a positive effect on the enterprise’s opportunity-seeking. Firstly, after the value chain horizontal decomposition, the entrepreneur can condense their core business, and focus on product improvement trials in advantageous links to improve product quality and production capacity and provide more possibilities for new R&D development and new order acquisition through reputation transfer.

The positive effects of horizontal decomposition of the value chain on enterprise opportunity-seeking are mainly shown in the following aspects. First, through the outsourcing of non-core businesses, the entrepreneur can focus on the competitive value creation link, and improve product quality and service quality through the release of technological advantages, which significantly improves product competitiveness and corporate reputation; this will provide more possibilities for new order acquisition and new market development.

*“Before the decomposition of the value chain, due to the many types of products and the different parameters required for each product, the process essentials of some products in the high-temperature crystallization link could not be accurately controlled, which eventually led to the unstable quality of these products.” (Interview with entrepreneur Tao Zhang)*

With the outsourcing of non-core businesses, the entrepreneur was able to focus on process improvement iterative experiments in the core technology segment. In particular, in the crystallization magnetization process, the entrepreneur has improved the product quality and qualification rate by improving the process of placing a number of different models of products in the high-temperature crystallization furnace, adding to the ratio of auxiliary materials and dynamic control of temperature.

*“After our product quality was improved, one customer increased the proportion of orders assigned to us from 30% to 70%... Two other customers put some of their new products in our company for pre-development, and the depth of our cooperation was strengthened. In short, after the value chain decomposition, our total orders increased rather than decreased. (Interview with entrepreneur Zhang ’s wife)”*

Secondly, business outsourcing and order decomposition make for a larger division of labor and collaboration between the entrepreneurs and the community members. This cooperation pattern accelerates the flow of knowledge and information sharing among enterprises, which helps the entrepreneur to deeply integrate technical advantages and market information. This facilitates the entrepreneur to develop more valuable products and services based on technical advantages to meet the individual needs of community members and obtain more profit streams for the enterprise. The related data structure is shown in Table 5.

Advantage-seeking. The impact of the horizontal decomposition of the value chain on the advantage-seeking of the enterprise is mainly demonstrated in the following aspects. Firstly, as the share of orders increases and the demand for raw materials continues to grow, enterprises can gain scale effects in procurement and obtain preferences from suppliers regarding raw material prices and billing deadlines. Second, the enterprise can also reduce logistics and storage costs by optimizing the frequency and quantity of procurement. Thirdly, the division of labor strengthens the trust relationship between the entrepreneur and the community members, the relationship governance and contractual governance effect are improved, and certain transaction cost advantages can be obtained.

Secondly, in the production process, with the optimization of order types, workers do not have to frequently switch between different types of raw materials and tools, which greatly improves production efficiency, as well as the articulation and continuity between all operation steps. The increased efficiency reduces the frequency of overtime work and ensures that employees can work on time. The optimization of work processes and working hours has improved employee job satisfaction. The health and well-being of employees in the workplace are further enhanced.

*“In the past, when orders were at their peak, I often worked overtime and sometimes night shifts, so I couldn’t take care of my family and children. Now this situation rarely occurs, basically all can leave work on time, every day work is very happy.” (Workshop staff interview)*

Moreover, the chance of mixing materials in production has been significantly diminished, thereby reducing the labor cost of repeated inspections of half-finished products. The improvement in efficiency and the increase in compensation are closely related, and employees’ well-being is satisfied with more organizational citizenship behaviors, which further improve the productivity of the company and reduce energy losses.

*“Nowadays, many veteran employees are very motivated and responsible, and they will take the initiative to suggest us anything that is not suitable or needs to be improved in their work. Many times, their suggestions can help the enterprise to improve efficiency and reduce energy consumption.” (Communication with production manager and technical manager in the symposium)*

The relevant data structure is shown in Table 5. Therefore, we propose:

**Proposition** **2.** *In the growth stage, the interaction between the entrepreneur’s Communitarian social identity and inventor and developer role identities enhances their entrepreneurial well-being and motivates the entrepreneur to complete the implementation of strategic entrepreneurial activities through behaviors such as resource orchestration and value chain decomposition.*

### 5.3. Expansion Stage of Entrepreneurship

#### 5.3.1. The Multiple Identity Types of Entrepreneurs in the Expansion Stage

The multiple identities presented by the entrepreneur during the expansion stage are complicated. The social identities are Darwinian and Missionary and the role identities are founder, inventor, and developer.

Around 2015, with a series of changes in the business environment, the company encountered some challenges and opportunities.

The challenges are mainly focused on the procurement of raw materials, where companies encounter problems such as untimely supply and unstable quality in the procurement of core raw materials. The opportunities are mainly reflected in a series of new national policies to support entrepreneurship from the national level to the local government level, for the new energy, new technology, and other innovative entrepreneurial projects from the land, financial, taxation, and other aspects of the designation of detailed preferential policies.

First of all, as the supply of raw materials in the crystal magnetic industry has been an oligopoly, the industry lacks a perfect bidding mechanism, and the bargaining power of customers in procurement is weak. In recent years, the prices of raw materials have gradually increased, and the profits of enterprises have shrunk year by year. In addition, the quality problems of raw materials emerge one after another, which affected the quality of products. This adversely affected the satisfaction of customers and the reputation of enterprises. Community members, especially some key customers, expect the entrepreneur to take effective measures to deal with this dilemma.

*“During that time, not only our company was troubled by the unstable quality of mid—and high-end strip, but also several peers often complained to me. Calm down, I also think, this matter always after the event is not a thing, not only need to spend a lot of manpower and financial resources to remedy, but also need to say a lot of good words, should find a way to solve the root. (Interview with entrepreneur Tao Zhang)”*

During this period, China’s State Council launched the “13th Five-Year Plan”, a comprehensive work program for emission reduction and energy conservation, which puts forward new requirements for the green transformation and development of enterprises. The program points out that governments at all levels should take energy conservation and emission reduction as an important grasp to optimize industrial structures, promote green, circular, and low-carbon development, and accelerate the construction of an ecological environment. Meanwhile, “mass entrepreneurship and innovation” is booming. The executive meeting of The Chinese State Council set up a 40-billion-yuan investment guide fund for venture capital investment in emerging industries, and the state issued specific support policies for enterprises’ green transformation, research and development of new materials and technologies, and other innovation and entrepreneurship projects. These entrepreneurial incentive programs provide new growth opportunities for many new energy, new materials, and high-tech companies. M Electronics is a high-tech company dedicated to new materials R&D, so it can also benefit from the relevant support policies.

*“For environmental considerations, we improved the heating furnace process and the placement and density of the cores in the furnace, and the heating time was able to be reduced by about 15%, and the product qualification rate was significantly improved; we also replaced the original disposable boxes with reusable boxes.” (Interview with entrepreneur Tao Zhang)*

Based on the current national entrepreneurship support policies, some peers and customers suggest the entrepreneur use his own technical advantages to R&D strip raw materials and eliminate the over-dependence on existing suppliers.

*“Once when chatting with a peer, he asked me why you don’t invest in your own plant to produce raw materials given that you have the technical advantages and R&D conditions to produce strips?” (Interview with entrepreneur Tao Zhang)*

The peer’s advice reminded the entrepreneur that this coincided with his long-standing desire to escape the passive situation in raw material procurement and inspired him to start a second business to build his own strip production line. As a result, the entrepreneur’s social identity has evolved into a Darwinian and Missionary hybrid social identity. 

In order to fully grasp the process of strip production, the entrepreneur also sought the opportunity to visit upstream strip producers and consult their technical staff. After returning, he conducted a series of experiments through knowledge absorption, and used the produced strip samples for trial production and testing magnetic core quality. After a year of exploration and experimentation, he had largely mastered the whole process of strip development and production. This process reflects the role identity of the developer.

Strip production is an energy-intensive industry that requires significant consumption of electrical power during the manufacturing process. The higher electricity price in Shanghai made it costlier for the entrepreneur to invest. In 2016, the entrepreneur received a message from a relative that an industrial park in his hometown of Shandong Province was attracting investment in new energy, new materials, and other innovative entrepreneurial projects, and qualified projects can benefit from the government’s preferential policies regarding plant leasing, energy, finance, and taxation. The R&D and production of nanocrystalline soft magnetic strip material are new material projects, which meet the requirements for applying to be stationed in this industrial park. After market investigation and comprehensive consideration, the entrepreneur decided to apply to invest in the industrial park to establish a raw materials production enterprise.

The entrepreneur then used his cousin’s social and political resources in his hometown to apply and undergo a government assessment, and was eventually approved for admission to the park. The entrepreneur started a new journey with the second venture. At this point, the entrepreneur presents the type of identity that is a Darwinian social identity and founder role identity.

Therefore, the multiple identities presented by the entrepreneur during this period are complicated. The social identities are Darwinian and Missionary and the role identities are founder, inventor, and developer.

#### 5.3.2. Multiple Identity Interaction and Entrepreneurial Self-Efficacy in the Expansion Stage

Entrepreneurial self-efficacy is the strength of an individual’s belief that he or she can succeed in various entrepreneurial roles and accomplish various entrepreneurial tasks [59]. It is the degree of confidence that entrepreneurs can use their knowledge to accomplish entrepreneurial tasks, and it is the basis of entrepreneurial motivation. In this study, we find that the interaction between multiple identities of the entrepreneur during the expansion period improves the self-efficacy of the entrepreneur.

First, the Darwinian social identity and the role identities of founder and inventor are the types of identities presented by entrepreneurs during the initial stage of M Electronics in Shanghai. The interaction of these identity types has enabled the entrepreneur to gain successful entrepreneurial experience and master some skills [60]. The successful journey helped the entrepreneur to clearly understand what needs to be done to successfully identify entrepreneurial opportunities and how to do it efficiently. Therefore, the entrepreneur has enough confidence to believe that he has the ability to solve the problems that he may face in the second venture, his experience also helps him to make a more reasonable allocation of his own resources in the process of opportunity development and utilization, and enhance his strength of belief in successfully completing various entrepreneurial tasks again.

*“When the Shandong R Electronics was created, some of the processes were similar to the original (the creation of M Electronics), we had some experience in process design, habit formation, rulemaking and resource deployment, etc., and also had the confidence to set up the enterprise to put it into production as soon as possible.” (Interview with entrepreneur Tao Zhang)*

Secondly, the entrepreneur’s Missionary identity clarifies the new goals. The developer identity enables the entrepreneur to provide kinetic support for the realization of his new goals based on his technological advantages, which prompts the entrepreneur to expand his business scope to seek sustainable growth through technological innovation and knowledge transfer, and ultimately achieves the social motivation and value orientation aspects of the Missionary identity. 

These identities support each other and synergize effectively to convince entrepreneurs of their ability to leverage their technical strengths to accomplish specific tasks expected of them by community members, and further enhance their entrepreneurial self-efficacy. The data structure is illustrated in Table 6.

#### 5.3.3. Multiple Identity, Entrepreneurial Self-Efficacy, and Strategic Entrepreneurship

Individuals with high entrepreneurial self-efficacy actively change or construct new environments and actively challenge the status quo rather than passively adapt to the current environment [61], which helps them to be more proactive in using the resources and support available in the business environment and facilitates the seek for entrepreneurial opportunities.

During the expansion period, they will also face certain resource constraints, when irrational factors such as personal experience, perceptions, and improvisation accumulated from the entrepreneurs’ previous Darwinian social identity and founder role identity will play an important role in the decision-making process of preparing for the establishment of Shandong R Electronics. Furthermore, higher entrepreneurial self-efficacy can make the entrepreneur more confident in his ability to create, obtain and orchestrate resources, he will prioritize them in product development and other aspects, and actively seek political support from the local government. When obtaining entrepreneurial support from the government, they will have more favorable expectations of entrepreneurial success and show stronger entrepreneurial intentions.

The ownership owned by the entrepreneur is itself a governance structure that highlights a state of entrepreneurship with little inherent restraint, and self-motivation and self-implementation of corporate governance are its typical characteristics. Based on identity interaction and entrepreneurial self-efficacy, the entrepreneur has more likelihood and chance to implement self-challenge and self-worth.

*“I don’t think we have too many restrictions. As long as we feel that the project has a good prospect and can bring long-term profits, we can make the decision to invest in the factory completely on our own, without worrying about other obstacles such as shareholders like other types of companies.” (Interview with entrepreneur Tao Zhang)*

In the second half of 2016, the entrepreneur integrated multiple resources and made strategic decisions regarding value chain vertical integration, and registered and established Shandong R Electronics in an industrial park in Shandong to operate the R&D and production business of amorphous nanoribbons. Currently, the products of Shandong R Electronics are not only supplied to Shanghai M Electronics but also supplied to some peers. The product feedback is good, the market size is also increasing steadily, and the company became profitable in 2019.

As opportunity-seeking and advantage-seeking are the two core dimensions of strategic entrepreneurship, we next focus on the impact of vertical integration of the value chain on strategic entrepreneurship from these two dimensions.

Opportunity-seeking. The vertical integration of value chains has a positive effect on opportunity-seeking for entrepreneurial companies. Firstly, value chain vertical integration enables entrepreneurs to expand the tacit innovation resources and the wide application of heterogeneous knowledge and facilitates opportunity search through the absorption, reorganization, and transformation of new knowledge, so that the technological advantages of the entrepreneur can serve new product development.

*“Strip production needs to melt the raw material at high temperature, the whole process requires excellent quality of machine parts, just one link failure of the whole production line to stop, we have been poorly controlled in the early stage. I tried to overcome the difficulties, in addition to learning from other people’s advanced experience, I also continued to consult information to start experiments to improve, and finally solved the problem by adjusting the machine mold spacing. After the spacing is adjusted, the strip can be rolled very thin, the product quality is also improved, and new customers and new orders gradually increased.” (Interview with entrepreneur Tao Zhang)*

Secondly, the vertical integration of the value chain expands the value network of the company, and the company has more opportunities and possibilities to acquire new customers and new markets. This will help the entrepreneur to be able to find new partners and build new partnerships based on new customer demands and trading relationships, and ultimately facilitate opportunity seeking.

*“We are engaged in an industry where companies are very close to each other, especially in product development where frequent communication is required. When we have a better corporate reputation, our customers will not only increase their share of buying our products, but also introduce new customers to us.”(Interview with Zhang ’s wife)*

Advantage-seeking. Value chain vertical integration also has a positive impact on advantage-seeking for companies.

First, with the extension of the value chain to the upstream raw material production chain, entrepreneurs are able to effectively control the quality and capacity of raw materials, which ensures the timeliness of raw material supply and enhances the timeliness of product supply.

*“We send raw materials from Shandong factory as we use them, which not only does not take up expensive storage space in Shanghai, but also reduces transportation and storage costs.” (Interview with entrepreneur Tao Zhang)*

Secondly, value chain extension enables companies to share business among multiple businesses and maximize resource utilization. Thirdly, the linkage between upstream and downstream businesses can realize resource interchange and internal transactions, which can reduce the cost arising from market transactions and generate economy-of-scope effects.

*“Before the establishment of the R company, we had very little bargaining power in raw material procurement and basically accepted suppliers’ prices passively; after the establishment of the R company, we had more bargaining power in raw material procurement to ensure fair prices.” (Interview with entrepreneur Tao Zhang)*

Thirdly, as entrepreneurs extend the value chain upstream to establish raw material production enterprises, there are no technical barriers and communication obstacles between upstream and downstream enterprises to ensure the effectiveness and convenience of communication, which can accelerate the transformation and application of technological advantages and improve the market competitiveness of the enterprise.

*“The best parameters of our products have been able to meet the standards of similar products in Germany, and we are now seeking international certification...... One of our products has now entered the procurement catalog of a state-owned enterprise and started mass supply.” (Technical director Mr. Liu)*

The data structure is illustrated in Table 6. Through the above discussion, we propose:

**Proposition** **3.** *In the expansion stage, the interaction between the entrepreneur’s Darwinian and Missionary social identities and founder, inventor, and developer role identities enhances the entrepreneurial self-efficacy and motivates the entrepreneur to complete the implementation of strategic entrepreneurial activities through behaviors such as resource creation and value chain vertical integration.*

## 6. Conclusions

In this study, based on strategic entrepreneurship theory, entrepreneurial role identity theory, and social identity theory, we explore the micro-driven mechanisms of strategic entrepreneurial behavior in technology-based family firms from the perspective of the dynamic evolution and interaction of multiple entrepreneurial identities. The findings provide a theoretical framework and action guidance for such firms to effectively address challenges and achieve sustainable growth in dynamic and uncertain contexts by implementing strategic entrepreneurial behavior.

The research found that entrepreneurial role identities and social identities evolve with the development of the entrepreneurial enterprise. At different stages of entrepreneurship, entrepreneurs’ multiple identity interactions affect their different entrepreneurial emotional states, which in turn influence their strategic decisions and entrepreneurial behavior. This ensures that entrepreneurs carry out resource acquisition, resource orchestration, resource creation, and other experimental innovation activities based on technological advantages to promote strategic entrepreneurial behavior in the company through horizontal decomposition and vertical integration of the value chain, and ultimately to achieve sustainable business growth and wealth creation.

In the initial stage of entrepreneurship, the entrepreneur’s main entrepreneurial motivation is to maximize economic benefits and improve their living conditions through the creation of a profitable business. However, due to survival pressure and highly uncertain resource constraints, the entrepreneur’s identity exhibits both a founder role identity and a Darwinian social identity. The interaction between the two identities enables the entrepreneur to maintain a high level of entrepreneurial passion, which enhances the entrepreneur’s ability to acquire resources, achieve technology transformation, and accelerate commercialization. It also ultimately promotes the implementation of strategic entrepreneurship in technology-based family firms.

In the growth stage, after a lot of work over previous years, the entrepreneur venture-journey goals are beginning to be realized, the operation of the company gradually finds the right track, they have stable customers, and the revenue of the company is gradually increasing. With the improvement of the working environment and living conditions, the entrepreneur gradually gains entrepreneurial well-being from his entrepreneurial activities. Meanwhile, the self-worth of the entrepreneur is also enhanced in the process of meeting the needs of community members and he receives more entrepreneurial well-being. As the opportunities for collaboration between the entrepreneur and community members increase, there are more interest connections and symbiotic relationships between them, so the social identity of entrepreneurs gradually evolves from a Darwinian identity in the early stage of entrepreneurship to a Communitarian identity. The entrepreneur will take the product needs of community members as the direction of his efforts, and look forward to obtaining the necessary information and support from the community for product quality improvement and new requirements development. Thus, his role identity gradually evolves into developer and inventor types. These multiple identities interact with each other to ensure that the entrepreneur can choose the areas or segments of interest for deeper cultivation. Through the division of labor and complementary strengths of community members, the company can gain competitive advantages and innovation opportunities for sustainable growth. 

In the expansion stage, the entrepreneurial motivation and self-evaluation basis of the entrepreneur changes as the business development environment changes. In order to reverse the passive situation of raw material procurement and enhance their industry position, the entrepreneur extends their corporate value chain upstream to the raw material production chain based on their relationship network and technological advantages. Thus, the social identity of the entrepreneur gradually evolves into Darwinian and Missionary social identities. The role of the entrepreneur has evolved to multiple roles of founder, developer, and inventor in order to raise new raw material production companies and develop new products. These identities interact to enhance the self-efficacy of the entrepreneur and motivate him to carry out strategic entrepreneurial behavior in the form of knowledge absorption, technology transfer, and business expansion. The vertical integration of the value chain not only contributes to the company’s opportunity-seeking by transferring technological advantages to new product development and product integration through iterative experimentation of product structure matching but also enables the company to enhance its industry position and cost advantage by expanding the application fields of technological advantages to realize the advantage seeking of the company.

The findings suggest that entrepreneurial initiative and multiple identity interactions play a key role in the implementation of strategic entrepreneurial activities in technology-based family firms. Entrepreneurs should dynamically adjust the salience of multiple identities according to their strengths and changes in the entrepreneurial context, which enhance their entrepreneurial passion, entrepreneurial well-being, and entrepreneurial self-efficacy at different stages of development. These different emotional states help entrepreneurs promote the implementation of strategic entrepreneurial activities, which can help them break through resource and institutional constraints to achieve their sustainable growth aspirations. The conclusion of this research provides new explanations for the micro-driven mechanisms of strategic entrepreneurial activity of technology-based family firms and also brings new inspiration to the strategic entrepreneurial practices of family businesses.

## 7. Discussion

Prior research has produced worthy insights into family firms, strategic entrepreneurship, and entrepreneurial identity. Based on these theories, the possible marginal contributions of this study include the following.

### 7.1. Theoretical Contribution

Firstly, this research promotes the study of strategic entrepreneurship within family businesses. Existing research mainly focuses on the relationship between resource orchestration and strategic entrepreneurship [7], intergenerational transmission of family firms and sustainability of strategic entrepreneurial activities [13], family member involvement and entrepreneurial strategic decision-making [10,12], and so on.

However, existing studies lack research on the micro-behavioral mechanisms of strategic entrepreneurship of family business entrepreneurs at different stages of business development. This research focuses on this problem in technology-based family firms. Based on the entrepreneur role and social identity theory [17,18,19,20], this study explores the behavioral mechanisms of strategic entrepreneurship in family firms through the perspective of entrepreneurial role identity and social identity interaction, which provides a new framework for the study of strategic entrepreneurial behavior and makes up for the lack of path evolution process analysis in existing studies on strategic entrepreneurship of family firms.

Second, our research responds to the concerns of some scholars regarding the integration of entrepreneurial role identity and social identity research. Most of the existing studies are only single and parallel studies on the role identity or social identity of entrepreneurs, and few studies integrate the two theories [18,21,23,25]. Moreover, there is a lack of research on the dynamic evolution and interaction of entrepreneurs’ multiple identities. This study responds to the calls of [18,21,25,32,34] for research on how entrepreneurs’ social identities and role identities change over time in serial entrepreneurship and explores why entrepreneurs’ social identities and role identities evolve during specific entrepreneurial phases, as well as answering the question of what impact does the interaction of entrepreneurial social and role identities have on entrepreneurial decisions and behavior. This study integrates entrepreneurial social and role identity theory to explore in depth how entrepreneurial identity evolves and interacts with firm development and how this interaction affects strategic decision-making and entrepreneurial behavior, which deepens and expands the field of research on entrepreneurial identity and also provides meaningful insight into the initiative of entrepreneurs in the implementation of strategic entrepreneurial behavior.

Third, the study enriches the theoretical research related to family firms. The topics of existing research on family firms are mainly focused on corporate governance [62,63], firm growth [64,65,66], intergenerational inheritance [13,65,66,67,68,69], family control [70,71], socioemotional wealth [7,72,73,74], and so on. Little research has been conducted on wealth accumulation and the continuous growth of family firms in complex and dynamic contexts, especially in the current environment of intertwined global economic downturn and the COVID-19 pandemic, and even less research has been conducted on how family firms can effectively respond to the shocks and challenges brought about by environmental changes. This study analyzes the impact of entrepreneur social and role identity interactions on different entrepreneurial emotional states, responding to scholars’ calls for family firm research that should deeply explore entrepreneurial emotions [75,76,77]. It was found that different emotional states of entrepreneurs affect their strategic decisions and behavioral choices, and influence the implementation of strategic entrepreneurial behavior in firms. This research also reveals the selection mechanism of strategic entrepreneurial behavior of technology-based family firms in special stages, which provides a useful reference for the wealth creation and sustainable development of firms under the challenges of complex dynamic situations and enriches theoretical research on family firms.

Fourth, this study also responds to some scholars’ proposals that manufacturing companies require horizontal integration through value networks, vertical integration through networked manufacturing systems, and end-to-end integration of engineering across the entire value chain in the context of the “Industry 4.0” strategy [78,79,80]. It provides theoretical insights for manufacturing companies to rethink and reshape their business models and implement effective strategic entrepreneurial behaviors to deal with the ever-changing customer demands and market turbulence.

### 7.2. Managerial Implications

First of all, entrepreneurial enterprises, at their different life cycle stages, seek resources from different actors with differing legitimacy evaluation criteria. This study finds that distinctive entrepreneurial identity structures are associated with entrepreneurial well-being, employee satisfaction, and stakeholder support, making it an important guarantee for entrepreneurs to access external resources that facilitate the synergistic effects of sustainable well-being and productivity in the corporate workplace. In management practice, startups should focus on workplace sustainability issues, especially on the sustainable well-being of employees and efficient productivity synergies. First, the company should strive to improve working conditions, such as working hours, work-family balance, nature of employment, meaningful work, etc. Second, companies should provide a work environment that brings out the potential and strengths of their employees, and enhance organizational citizenship through the realization of employee values. Third, the company should strive to enhance the synergy between sustainable well-being and efficient productivity through incentives and resource orchestration to achieve workplace sustainability and sustainable corporate development. Third, companies should strive to enhance the synergy between sustainable well-being and efficient productivity through incentives and resource scheduling to achieve workplace sustainability and corporate sustainability. Specifically, startups should pay attention to the dynamic function of senior managers’ identities, and pay attention to the heterogeneity and interaction of managerial identities of different positions in the entrepreneurial process to give full play to the subjective initiative of core employees, especially the evolution and interaction between different identities of entrepreneurs, and adopt different resource orchestration methods to carry out opportunity search and advantage search based on the core competitive advantages of the enterprise to achieve wealth creation and sustainable development of the enterprise.

Second, entrepreneurs should be skillful in finding the prototype of entrepreneurial identity to provide a reference for the construction of self-identity. Moreover, entrepreneurs should also try to delicately combine their strengths, the characteristics of opportunities, and market traits in order to choose the areas that can highlight their strengths for entrepreneurship, and through a proper identity narrative to obtain internal and external relationship network entrepreneurial support. In the initial stage, entrepreneurs should strive to seek support from potential stakeholders, form entrepreneurial teams, and obtain resource support, then organically integrate competitive advantages and external resources to quickly complete enterprise creation, customer acquisition, and initial product development to shorten product development time and quickly obtain economic benefits. In the growth stage, entrepreneurs should carry out knowledge absorption, technology embedding, and iterative testing activities through resource integration and other activities to improve product quality and competitive advantages, as well as to boost the company’s opportunity-seeking and advantage-seeking through the optimal allocation of key resources. During the expansion period, entrepreneurs can carry out innovative activities based on their competitive advantages and expand the application of their dominant resources according to the dilemmas and changes in the situation encountered in the development of the company. Through new product development and new market exploration, the entrepreneur can enable the company to obtain sustainable development.

Third, government departments such as policy-makers, when designing policies related to innovation and entrepreneurship, should not only consider factors such as products, technology, and scale of startups but also the stage characteristics of startups, paying special attention to the competitive advantages and identity traits of business managers, and develop more stimulating fiscal and credit policies. Positive policy guidance and incentives can promote the transformation and upgrading of enterprises and the marketization process to guide the healthy and sustainable development of the whole industry.

Last but not least, under the strategic background of “Industry 4.0” and “Made in China 2025”, this study has some practical insights for other technology-based firms or other family firms, especially some new energy and new technology manufacturing enterprises for the purpose of upgrading and transforming their businesses. Enterprises should fully understand the important role of product technology research and development and adopt different ways of product technology R&D to promote the intelligent upgrading of enterprises in different development periods. In the initial stage, entrepreneurs should focus on technology application and transformation based on product demand, and complete the production and sales of self-developed exhibits. In the growth period, entrepreneurs take iterative product upgrades and collaborative innovation based on market demand and community needs and strive to provide customers with integrated product and service solutions as a whole. In the expansion period, entrepreneurs should focus on the transformation and upgrading of intelligent manufacturing capabilities that match the development of the enterprise through analysis and prediction, remote operation, and technology extension based on the knowledge of national macro policies, and not blindly expand.

### 7.3. Limitations and Future Research

Despite the above-mentioned contributions, this research also has two limitations that suggest future research directions. First of all, our study was conducted based on a single case, and the external validity of the obtained propositions and theoretical models needs to be tested in depth. These theoretical propositions can be consolidated and refined through multiple case studies or large-sample statistical empirical studies in the future to further verify the validity of the findings. Second, in this research, we selected technology-based family firms as the sample case, whether the findings and the theoretical model concluded can be applied to other types of firms needs to be explored in depth in the future to improve the generalizability of the research results.

## Figures and Tables

**Table 1:** Details of data collection.

Date	Days	Data Source	Content
March 2020	3	Corporate website homepage; Network Public Information	Obtain relevant information on the subject and formulate a research plan
April 2020	0.5	Open Interviews with entrepreneur	Negotiate the specific arrangements for the interview
May 2020	2	Interviews with entrepreneur, family members, and employees; archival material	In-depth interview with entrepreneur, family members, and employees; Obtain some documentation.
August 2020	2	Onsite observation; Staff Interviews	Conducting on-site observations and conversation
December 2020	1	Publicity material; Conversation; Stakeholder Interviews	Communicated with the employees Checking annual reports; Interview with entrepreneur
June 2021	1	Employees of the company; Supplier and customers	Provides feedback to the enterprise; Conduct in-depth interviews
September 2022	1	supplementary interview	Communicate with the entrepreneur to learn about the current situation in the company.

**Table 2:** Representative Quotations of Open Coding.

Representative Quotations	First-Order Concepts
“I want to use the skills I’ve learned to create a profitable business, make entrepreneurial profits, and improve the living conditions of my family.” (Interview with Tao Zhang)	Building profit firms
“He outsources the orders he is not good at to us, and we introduce him to some new customers and orders.” (Stakeholder Interviews)	Complementary advantages
“... Resources can be shared between upstream and downstream companies in terms of technology upgrading, market development, customer relationship maintenance, advertising and marketing, and cost sharing.” (Interview with Tao Zhang)	Cost allocation
“Manager Han and others also helped me collect data and made suggestions for improvement. We improved the technical standards of key links, and the product parameters index and market competitiveness have been improved significantly.” (Interview with Tao Zhang)	Product improvement experiments

**Table 3:** Example of Data Structure.

First-Order Concepts	Second-Order Themes	Aggregate Dimensions
Building profit firms Formation of team	Founder	Entrepreneur Role Identity
New product design New market development	Developer	
Cost allocation Efficiency advantage	Advantage-seeking	Strategic Entrepreneurship
Get new customers New products R&D	Opportunity-seeking	
Wholeheartedly involved A sense of pleasure	Positive emotions	Entrepreneurial Passion
Self-identification Recognition by others	Identification	

**Table 4:** Example of the Data Structure in the Initial Stage.

The Representative Quotations for First-Order Concepts	Second-Order Themes	Aggregate Dimensions
Building profit firms “I want to use the skills I’ve learned to create a profitable business, make entrepreneurial profits, and improve the living conditions of my family.” (entrepreneur)” Pursue private economic goals “At that time, my main motivation was to make money through the application of technology, and ultimately leading to personal wealth accumulation.” (entrepreneur)	Darwinian identity	Entrepreneur identity
Establishing funding “In addition to some savings from work, I borrowed some funds from relatives and friends, a total of more than 600,000.” (Interview with Zhang) Finding employees “I brought over 10 people from my hometown, and my family also participated. I invited 6 more former colleagues to join me in the business.” (Interview with Zhang)	Founder	
Cost advantage “They (family members) worked in the company since its inception, which saved a lot of labor costs; the family members and Manager Han followed me for a long time, which ensured the stability of the company’s core staff and saved a lot of recruitment and training costs by avoiding frequent recruitment of staff.” (Interview with Zhang) Efficiency advantage “Stakeholders are collaborative and stay in touch with us to provide timely feedback on data, so that we can make improvements quickly and significantly reduce the time to commercialize our products.” (Zhang’s wife)	Advantage -seeking	Strategic entrepreneurship
Acquisition new customer “Our customers use our products and give them high praise. We developed many new customers based on the acquired market reputation.” (Interview with Zhang) R&D new product “My wife’s brother, manager Han and other staff were engaged in the development and testing of the new product. Our customers gave us a lot of feedbacks on the samples, and we made improvements according to the customers’ suggestions, the product was soon finalized and mass produced.” (Interview with Zhang)	Opportunity-seeking	
Wholeheartedly involved “I have spent all my time on product development and technology application and am very keen to shorten the process of product commercialization as soon as possible.” (Interview with Zhang) A sense of pleasure “To be frankly, I feel happy to be able to use the skills I have to do what I like, even if I am tired.” (Interview with Zhang)	Positive emotions	Entrepreneurial passion
Self-identification “I am confident to create the enterprise and create the product because I visit other enterprises and I am more familiar with their production process.” (entrepreneur) Recognition by others “We know him very well, he has excellent skills... and do things more down-to-earth, follow him to do venture together feel promising.” (Zhang’s family members)	Identification	

**Table 5:** Data Structure in the Growth Stage (partial).

The Representative Quotations for First-Order Concepts	Second-Order Themes	Aggregate Dimensions
Division of labor and collaboration “From the initiation of our business to now, many of our peers have grown up with us. We are very familiar and understand each other’s strengths and core products...... For some orders that we are not skilled at, we will transfer it to our peers to do. They encounter orders they are unskilled at, they will also transfer to us.......” (Zhang’s wife) Cooperative innovation “Sometimes we will cooperate with some peers who have good relationship to bid for some grand orders, through order decomposition and deep cooperation to complete together. In the new product development, customers will give us some valuable feedback to help us improve product design and enhance product quality.” (Tao Zhang)	Communitarian identity	Entrepreneur identity
Recognizing invention “The customer proposed product requirements and parameter criteria, I developed them according to the requirements and soon produced samples.” (Tao Zhang) Developing new demands “Once I visited a customer’s company and found that they needed to add copper wire and packaging boxes after purchasing our products, I told them that they could add this link at the end of our production line to help them solve this problem” (Tao Zhang)	Inventor	
Procurement scale effect “As our share of raw material purchases increases, certain price and billing discounts are available at our suppliers. When the market for a particular raw material is tight, suppliers will also give priority to securing our needs.” (Zhang’s wife) Efficiency advantages “With the optimization of order categories, the efficiency of sorting and shipping our goods is now significantly improved, and we no longer have to worry about sending the wrong products.” (warehouse manager Wang).	Advantage-seeking	Strategic entrepreneurship
New market expansion “When our product quality and on-time delivery improved significantly, one customer increased the share of orders allocated to us from 30% to 70%, and others entrusted some new products to us for research and development.” (Zhang’s wife) Development of new customer needs “We provide product integration services to some of our customers based on their new needs. That is, we integrate components they provide such as transformers, stabilizers, bases, etc. with our cores and adapt the parameters to meet their needs.” (Tao Zhang)	Opportunity-seeking	
The experience of satisfaction “Now the profitability of the enterprise has been enhanced, and the living condition of my family has been improved. I feel very satisfied.” (Tao Zhang) Do the things I want “The things you want to do and the things you can do are consistent, so you can focus on technology development without worrying about the tedious management affairs.” (Tao Zhang)	Satisfaction	Entrepreneurial well-being
Entrepreneurial Success “Now the business is running very well, there are fixed customers, the scale is gradually increasing, and the economic benefits are increasing year after year.” (Tao Zhang) Personal development “I feel happy to work with my boss... I have met a lot of like-minded people and my technical and management skills have been greatly enhanced.” (Production Manager Han)	Self-actualization	

**Table 6:** Data Structure in the Expansion Stage (partial).

First-Order Concepts and Representative Quotations	Second-Order Themes	Aggregate Dimensions
Responding to community concerns “At that time, I was advised by my peers to R&D and produce strips to cope with the oligarchizing of the supply market…, the state issued a policy to support new material projects, and our project was qualified.” (Tao Zhang) Energy conservation and emission reduction “During product transportation, we use recyclable boxes; we have improved the production process of our heating ovens, and we treat wastewater in a harmless manner to reduce the discharge of pollutants.” (Tao Zhang)	Missionary identity	Entrepreneur identity
Market development “Using relatives in the local social resources and government relations, we have developed two more large customers, the market scale continues to increase” (Tao Zhang) Creating value “The value chain has been extended to the upstream material production segment. We have successfully produced strips by our own technology, and the added value of raw materials is now higher than the products of Shanghai companies.” (Tao Zhang)	Developer	
Product Quality advantage “The best parameters of our products have been able to meet the standards of similar products in Germany, and we are now seeking international certification...... One of our products has now entered the procurement catalog of a state-owned enterprise and started mass supply” (Technical Director Mr. Wang) Improve bargaining power “Before the establishment of the R company, we had very little bargaining power in raw material procurement and basically accepted suppliers’ prices passively; after the establishment of the R company, we had more bargaining power in raw material procurement to ensure fair prices.”(Procurement manager Mr. Li)	Advantage-seeking	Strategic entrepreneurship
New markets development “We are engaged in an industry where companies are very close to each other, especially in product development where frequent communication is required. When we have a better corporate reputation, our customers will not only increase their share of buying our products, but also introduce new customers to us.” (Zhang’s wife) Provision of new services “We now offer new product integration services for two customers, one is magnetic core integration and the other is inductor integration……We are now offering an increasing variety of new services to our customers.” (Tao Zhang)	Opportunity-seeking	
Prior entrepreneurial experience “After all, I have had a successful entrepreneurial journey, accumulated a lot of experience and social relations over the years, so I am more confident about the second venture.” (Tao Zhang) Technology advantage “I was already skilled in developing and producing strips and was able to produce samples of excellent quality.” (Tao Zhang)	Self-confidence intensity	Entrepreneurial self-efficacy
Risk-taking “At that time, the investment of several million to build the plant was risky, and all the earnings of these years were used as investment.” (Tao Zhang) Action ahead “Before investing in the factory, we had already produced number of samples and tried them inside our own workshop for iterative trials based on feedback.” (Tao Zhang)	Entrepreneurial endeavors	

## Data Availability

Not applicable.
